# 

**Published:** 1892-07-02

**Authors:** 


PRICE TWOPENCE.

No. 301, Vol. XII.—July 2nd, 1892.

Registered at the General Post Office as a Newspaper for transmission in the United Kingdom and Abroad.]

WITH TWO EXTRA SUPPLEMENTS.

Per Annum, 8s. 6d., post free.

Monthly Parts, 6d.; post free, 8d.

Ditto per Annum, 8s., post free.

### THE HOSPITAL

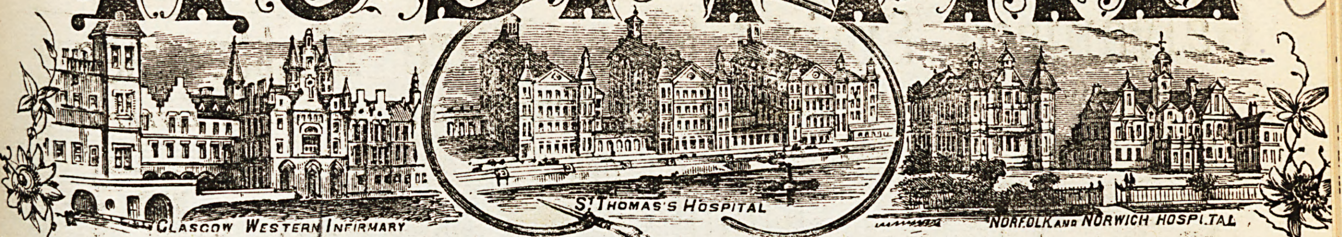

WITH EXTRA NURSING SUPPLEMENT.

No. 301. Vol. XII.] SATURDAY, JULY 2nd, 1892. [TWOPENCE.

